# *Rickettsia* species in *Dermacentor reticulatus* ticks feeding on human skin and clinical manifestations of tick-borne infections after tick bite

**DOI:** 10.1038/s41598-023-37059-3

**Published:** 2023-06-19

**Authors:** Julia Koczwarska, Agnieszka Pawełczyk, Justyna Dunaj-Małyszko, Justyna Polaczyk, Renata Welc-Falęciak

**Affiliations:** 1 Department of Parasitology, Faculty of Biology, University of Warsaw, Miecznikowa 1, 02-096 Warsaw, Poland; 2 Department of Immunopathology of Infectious and Parasitic Diseases, Medical University of Warsaw, Pawińskiego 3C, 02-106 Warsaw, Poland; 3 Department of the Infectious Diseases and Neuroinfections, Medical University in Białystok, Żurawia 14, 15-540 Białystok, Poland

**Keywords:** Parasitology, Parasitic infection, Epidemiology

## Abstract

*Dermacentor reticulatus* ticks are sporadically removed from human skin and therefore the medical consequences of their feeding are neglected compared to *Ixodes ricinus.* We investigated the prevalence of pathogens in *D. reticulatus* removed from human skin and possible clinical manifestations suggestive of tick-borne diseases after a tick bite. A total of 2153 ticks were studied and of these only 34 were *D. reticulatus*. The mean prevalence of *Rickettsia* in *D. reticulatus* was 50.0% and *R. raoultii* was identified in 82.4% of infected *D. reticulatus* ticks. We confirmed the first case of *R. aeschlimannii* infection in *D. reticulatus* ticks. Among participants bitten by *D. reticulatus*, 13.3% reported reddening around the tick bite site and flu-like symptoms, including lymphadenopathy and 3.3% reported eschar on the tick site bite. All of the participants with flu-like symptoms after tick removal were bitten by ticks infected with *R. raoultii.* The results of this study indicate that even though *D. reticulatus* ticks bite humans sporadically, pathogenic *Rickettsia* have a remarkably high prevalence in this tick species*.* We can expect that the incidence of tick-borne lymphadenopathy might increase with the reported expansion of the *D. reticulatus* into new areas and its growing abundance in Central Europe.

## Introduction

Ticks, right after mosquitoes, are the most important vectors of pathogenic microorganisms for the veterinary and public health interest. The main vector for tick-borne pathogens in Europe is the common tick *Ixodes ricinus* and the whole of Europe is considered to be an endemic region for this tick species^1^. Previous studies have shown that *I. ricinus* typically constitute 90–100% of all ticks removed from humans in Europe and that nymphs are the most commonly detected life stage associated with zoonotic pathogen transmission^2–4^. The geographical range of *Dermacentor reticulatus* in Europe is discontinuous and the spreading of these ticks is believed to be associated with the loss of forest area^5^. It also seems that the temperature and length of the growing season create no barrier for the spread and distribution of *D. reticulatus* in the territory of Poland^5^. *Dermacentor reticulatus* ticks, mainly adults, can bite humans and are sporadically removed from human skin, therefore the medical consequences of their feeding are neglected compared to *I. ricnus*^3,4,6–9^. Distribution of the pathogen in European *D. reticulatus* tick populations seems to be very uneven^10,11^. *Dermacentor reticulatus* is the main vector of *Babesia canis*, the aetiological agent of canine babesiosis, as well as spotted fever group (SFG) rickettsiae with *Rickettsia raoultii* and *R. slovaca*, recognized as causative agents of rickettsioses with typical lymphadenopathies, called tick-borne lymphadenopathy (TIBOLA) or *Dermacentor*-borne necrosis erythema and lymphadenopathy (DEBONEL), which are widespread in Eurasia^11,12^. *Rickettsia helvetica*, which causes milder symptoms, was also reported from *D. reticulatus* ticks^13^. Only several cases of TIBOLA/DEBONEL have been recorded in Poland so far^14,15^. Up till now the presence of *Borrelia burgdorferi* s.l., *Anaplasma phagocytophlium*, *Bartonella* spp. *Coxiella burnetti*, *Francisella tularensis,* TBEV, Omsk hemorrhagic fever virus have been also confirmed in *D. reticulatus* suggesting a possible role of this tick species in the life-cycle and transmission of these pathogenic microorganisms^13,16^. However, the low prevalence of some of these pathogens pose questions about the status of *D. reticulatus* as their vector.

The majority of published papers have focused on the pathogens prevalence in questing *D. reticulatus* ticks or engorged ticks collected from wildlife or domestic animals, mainly dogs. In rare situations, especially with a lack of other vertebrates in the area, people are chosen by *Dermacentor reticulatus* as a source of blood^17^. *Dermacentor reticulatus* ticks generally constitute less than 3% of all ticks removed from human skin^3,4,18^ and therefore its role in pathogen transmission or medical consequences of a tick bite is often ignored. The studies about the *D. reticulatus* infestation in humans and the risk of clinical manifestation after tick bites, even if the rate of incidences is low, may be crucial among others for at-risk personnel, such as foresters, hunters, soldiers, and farmers. It is worth noting that it is a very expansive species, resistant to even hard environmental conditions, and year by year more often detected in cities^17,19^. As far as we are aware, no studies assessing the risk of possible symptoms of tick-borne infection in humans after *D. reticulatus* feeding have previously been carried out in Europe. The main aims of the present study were to investigate the prevalence of tick-borne pathogens in *D. reticulatus* ticks removed from human skin and possible symptoms and clinical manifestations suggestive of tick-borne diseases after a tick bite.

## Results

### Dermacentor reticulatus ticks

A total of 2153 ticks removed by participants during 2021–2022 could be further investigated, and of these only 34 (1.6%) were *D. reticulatus*. Of the 34 feeding *D. reticulatus*, 2 (5.9%) were nymphs, 25 (73.5%) were females, and 7 (20.6%) were males. The molecular studies have confirmed the morphological identification of two *D. reticulatus* nymphs. The nucleotide sequences of the *cox1* gene fragment of tested nymphs were identical and highly similar (99.9%; 778/779) to the sequence originally obtained from *D. reticulatus* in Czech Republic (OM142141;^20^) and Novosibirsk region in Russia (OM867328). Most *D. reticulatus* ticks were collected from September to November (21; 61.8%). Only 6 (18.2%) ticks were removed in July and August, however, the majority of them (5 out of 6) were collected between the 20th and 31st of August. The rest of the ticks (7), including two nymphs, were brought to the laboratory in May and in the first half of June.

**Figure 1:**
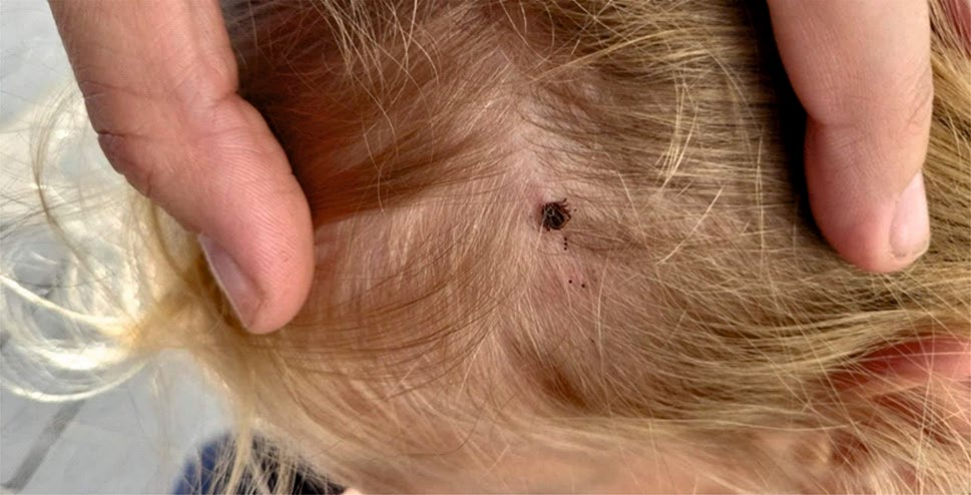
*Dermacentor reticulatus* female feeding on the head (scalp) of a 5-years-old girl.

**Figure 2:**
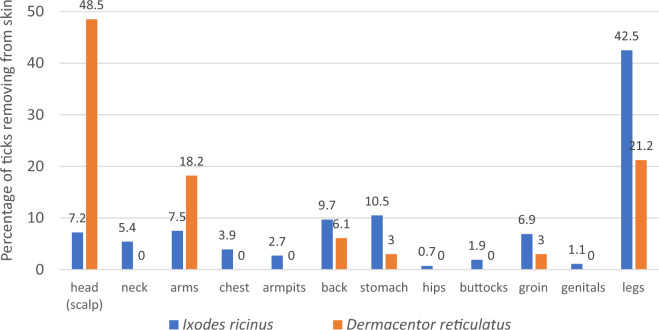
Anatomical distribution of *Ixodes ricinus* (n = 837) and *Dermacentor reticulatus* (n = 33) ticks reported by tick-bitten participants (n = 690).

Only one *D. reticulatus* tick was removed from the skin of each participant (n = 34). The data about among others sex and age of participants, anatomical location of tick bites, and flu-like symptoms after tick bite were collected and then analysed on the basis of filled-out questionnaires at enrolment (33 [97.1%] of completed questionnaires) and after 8 weeks (30 [88.2%] of completed questionnaires). Twenty-four (72.7%) out of tick bites were recorded in suburban and rural areas. Statistical analysis of the anatomical location of tick bites revealed significant differences between *I. ricinus* and *D. reticulatus* ticks (χ_11_^2^ = 52.5; p = 0.0001). Nearly half of attached *D. reticulatus* ticks (48.5%; 16/33; 95% CL 32,2–65,1%) were found on the head among the hair (scalp) whereas only 7.2% (60/836; 95% CL 5.6–9.1%) of *I. ricinus* were there recorded (Figs. 1 and 2). Similar number of *D. reticulatus* ticks were attached to the head of women and men (9/16 vs. 7/16). *Dermacentor reticulatus* ticks were also found more frequently on the arms compared to *I. ricinus* (18.2%; 6/33; 95% CL 8.0–33.7% vs. 7.5%; 63/836; 95% CL 5.9–9.5%). The reverse trend was observed on the legs where 42.5% (356/836; 95% CL 39.2–45.9%), and 21.2% (7/33; 95% CL 10.0–37.2%) of *I. ricinus* and *D. reticulatus*, respectively, were removed (Fig. 2). Single *D. reticulatus* ticks were also found on the stomach, back and groin.

### Description of the Dermacentor reticulatus bitten participants

More women than men participated in this study (22/33; 66.7% vs. 11/33; 33.3%) and the mean age of participants was 34 years (range 4–62). The majority of the study individuals (90.9%; 30/33) have declared that they did not use chemoprophylaxis (repellents) against ticks. However, 78.8% (26/33) of all participants have admitted wearing protective suits (i.e. long sleeves and legs, headgear, tucking the pants into long socks) when they were bitten by ticks. Three out of 33 participants (9.1%) were blood donors.

### Tick-borne pathogens prevalence

No *A. phagocytophilum*, *B. burgdorferi* s.l., *Babesia* spp., and *N. mikurensis* DNA were detected in *D. reticulatus* ticks. The mean prevalence of *Rickettsia* was 50.0% (17/34), without non-significant difference between tick development stage (nymphs: 1/2, 50.0%; females: 12/25, 48.0%; males: 4/7; 57.1%; p = 0.56), year of study (2021: 9/17, 52.9%; 2022: 8/17, 47.1%; p = 0.732) or urban/rural areas (urban: 4/10, 40.0%; rural: 13/23, 56.5%; p = 0.382). *Rickettsia* species typing was performed on the basis of the *gltA* gene fragment (730-bp product); all positive PCR samples were sequenced. Alignment and BLAST-NCBI analyses revealed the presence of three *Rickettsia* species. Of the 17 isolates, 14 (82.4%) were identical to *R. raoultii* isolated from *D. reticulatus* in our previous study^16^. Two isolates were identified as *R. helvetica* with a 100% of similarity level with *R. helvetica* from *I. persulcatus* in Novosibirsk (KU310588) and from *I. ricinus* in our previous study (MH018977;^21^). One nucleotide sequence was identical with *R. aeschlimannii* (KU961540;^22^) originally isolated from *Hyalomma marginatum* in the Crimean Peninsula (Supplementary File S1). To confirm the *Rickettsia* species identification, the outer membrane protein B (*ompB*) gene fragment (765-bp product) of randomly selected isolates of each species (*R. raoultii*, *R. helvetica* and *R. aeschlimannii*) was amplified and sequenced. The nucleotide sequences of the *ompB* gene fragment were, respectively: (i) identical with *R. raoultii* from *D. reticulatus* in Kaliningrad (ON191725) and in Germany (HQ232278;^23^); (ii) highly similar (99.7%; 763/765) to *R. helvetica* from *I. ricinus* in Germany (HQ232251;^23^) and from *I. persulcatus* in Russia (KU310591); (iii) *R. aeschlimannii* from *Hyaloma marginatum* in the Crimean Peninsula (KU961544;^22^).

### Self-reported non-specific symptoms among the participants after 8 weeks of tick bite

In total 30 study participants removed *D. reticulatus* ticks of which 1 (3.3%) reported reddening around the tick bite site, 2 (6.7%) reported flu-like symptoms and 4 (13.3%) reported both of them. One participant (3.3%) reported eschar (‘tache noir’) diagnosed by a general practitioner (GP) on the *R. raoultii*-infected tick bite site. Reddening at the bite site did not show the pattern of *erythema migrans* in any of the cases on the basis of GP diagnosis. Flu-like symptoms included fever (n = 6), headache (n = 6); malaise (n = 3), fatigue (n = 3), muscle pain (n = 2) and lymphadenopathy on the neck (n = 2). Interestingly, all of the participants who reported flu-like symptoms after tick removal were bitten by *D. reticulatus* ticks infected with *R. raoultii* (χ^2^_1_ = 8.85; p = 0.003). No significant differences were observed in the frequency of reddening at the bite site presence among participants bitten by *Rickettsia*-infected and non-infected ticks (p = 0.176).

## Discussion

To our knowledge, this is the first study on the risk of possible symptoms suggestive of tick-borne infection in humans after *D. reticulatus* removal from the skin. The main limitation of our study was the low number of collected ticks, however, adults *D. reticulatus* feed mainly on wild ungulates whereas immature forms—on rodents, and therefore they are sporadically removed from human skin^24^. Nevertheless, during the last two decades, an increasing number of studies has reported significant habitat expansion of *D. reticulatus* ticks in several European countries, including Poland^10,17,25,27^. The geographical range expansion of *D. reticulatus* ticks is concerning in light of their vector potential and high prevalence of tick-borne pathogens i.e. *Rickettsia*.

In our present and previous studies^3^, *D. reticulatus* ticks showed a bimodal activity pattern, with the highest density in May–June (the first half) and September–November, whereas only single ticks were collected in the summer (end of August), which is typical for this tick species^28,29^. Since insectivores and small rodents (mostly voles) are mainly hosts for larvae and nymphs and for adult ticks—medium-sized mammals (carnivores, sheep, goats, deer, cattle, and European bison)^24^, the risk of being bitten by *D. reticulatus* is significantly higher on suburban and rural areas. However, this pattern is likely to be characteristic also for *I. ricinus* tick species^30^. In our study women removed a greater proportion of *D. reticulatus* ticks compared to men which might reflect morphological, behavioral, and physiological differences between men and women^31^. Wilhelmsson and co-authors^31^ have suggested that men and women can differ in their capacity to rapidly detect the tick on the skin due to more hairy skin of the men and/or their grooming behavior. Moreover, it has been shown that women have a higher risk perception concerning tick bites that could influence the protective behaviour^32,33^. Nonetheless, in our study the majority of the participants (> 90%) have declared not to use chemoprophylaxis against ticks. It could be the result of disliking the idea of applying a chemical to bodies (especially in case of children), perceiving repellents as unsafe, and the frequency (usually every 4–6 h) or method of application of tick repellents^33,34^. Simultaneously, almost 80% of respondents have admitted wearing protective suits when they were bitten by ticks, which seems to be an insufficient protection measure.

It is believed that humans are likely to be bitten by different tick species in potentially various anatomical sites and in varying seasonality^2,35^. In our study, results should be interpreted with caution due to significant discrepancies in the number of groups of both tick species, however some differences in ticks' most likely location could be observed. All *D. reticulatus* life stages were found significantly more frequently on the head (scalp) compared to *I. ricinus* ticks. Despite containing less than 10% of the surface area of the human body, the head was identified as the attachment point of almost 50% of all *D. reticulatus* ticks. The authors of previous studies^2,36^ have suggested that especially children are prone to be bitten by ticks on the head and/or neck due to behavioral (frequent enter vegetation while playing) and physiological (head/neck on the level of vegetation with questing ticks) differences between adults and children. In our study only 5 *D. reticulatus* ticks (15.2%; 5/33) were removed from children under 15 years, however the majority of them (4/5; 80.0%) were collected from the head (scalp). Therefore, it seems that adult *D. reticulatus* ticks, parasitizing mainly medium-sized mammals, prefer the hairy skin of the head. It also corresponds with the fact that the majority (> 93%) of patients affected by *Dermacentor*-Borne-Necrosis-Erythema-Lymphadenopathy (DEBONEL) were bitten on the scalp^37^. According to Hart and co-workers^38^, this is also clear evidence of the climbing behaviour of *Dermacentor* ticks. It is likely that hair protects them from being immediately detected and removed by animal hosts, obscuring them until they can feed extensively^38^. It is also worth noting that the tick site attachment to the human body might be of clinical importance^31^. Significantly greater proportion of neurological manifestation of Lyme borreliosis (LB) among patients who had been bitten on the head or neck was shown than among LB patients bitten on other parts of their body^39^. Therefore, understanding ticks' most likely location can be useful for removing them to minimise pathogens transmission or diagnostically to confirm the presence of the tick.

Despite the fact that *D. reticulatus* ticks constituted less than 3% of all ticks collected from human skin (1.6% in our study)^2,40^ they play the key role as the *Rickettsia* vectors. In our study prevalence of *Rickettsia* in *D. reticulatus* ticks was 50% whereas no *A. phagocytophilum*, *B. burgdorferi* s.l., *Babesia* spp. and *N. mikurensis* DNA were detected. Our results of *Rickettsia* prevalence correspond with our previous study^16^ as well as with other data from Poland^41,42^, Germany^43^, Hungary^44^ and Slovakia^11^. Not surprisingly, the majority of all *Rickettsia* isolates (14 out of 17) were identified as *R. raoultii*. *Rickettsia helvetica* is identified rarely in *D. reticulatus* compared to *R. raoultii*^45,46^ and *I. ricinus* ticks serve as the main vector for this pathogen species. According to our best knowledge, we have confirmed for the first time the presence of *R. aeschlimannii* in *D. reticulatus* ticks. *Rickettsia aeschlimannii* was described in 1997 as the new spotted fever group *Rickettsia* associated with *Hyalomma marginatum* ticks^47^. *Rickettsia aeschlimannii* is an emerging human and animal pathogen, reported from various ticks in Europe and Africa, including several *Hyalomma* spp. ticks collected from migrant bird species^33,48^, which might explain the presence of this pathogen in ticks collected in Poland. The first human infection caused by *R. aeschlimannii* was reported for a French patient who became ill after returning from Morocco and who exhibited symptoms similar to those of Mediterranean Spotted Fever^48^. First human cases of *R. aeschlimannii* infection were recently noted in Russia and China^49,50^. In our study, no symptoms of tick-borne infection were declared by a participant who was bitten by *R. aeschlimannii*-infected tick. However, the role of *D. reticulatus* as a vector of *R. aeschlimannii* in Europe need further, urgent investigation.

Several cases of DEBONEL/TIBOLA have been described so far in Europe, including Poland^14,15,51–54^. At the site of *Dermacentor* tick bite, a high percentage of patients develop an inoculation eschar (necrosis) surrounded by an erythema and regional enlarged and painful lymphadenopathies^55^. In 40% of patients affected by DEBONEL fever was also observed^37^. Here, the flu-like symptoms (fever, headache, malaise, fatigue and muscle pain) were noted in 6 out of 30 study participants (20%), including two respondents who simultaneously reported lymphadenopathy on the neck. All participants who declared flu-like symptoms (including lymphadenopathy on the neck) after tick removal were bitten by *D. reticulatus* ticks infected with *R. raoultii* what might suggest the *Rickettsia* infection. However, further serological studies are needed. In our study, 2 out of 3 blood donors were bitten by *R. raoultii* infected ticks and one of them reported flu-like symptoms. Several tick-borne pathogens can potentially be transmitted through blood transfusion, i.e*. Anaplasma phagocytophilum* or *Babesia microti*^56^. Many tick-borne microorganisms are located intracellularly, which is an excellent condition for transmission by transfusion^56^. The risk of transfusion-transmitted *Rickettsia* is unknown, however, single cases of *R. rickettsia* and *R. parkeri* have been described so far^57,58^. Lucchese and co-authors^59^ have demonstrated that *R. conorii* was able to remain viable in dog blood stored until 35 days. Therefore further studies about the risk of transfusion-transmitted *Rickettsia* infection are essential, especially if we take into account that blood recipients are often immunosuppressed^60^.

In our study in the case of one participant, *Rickettsia* infection was diagnosed by GP on the basis of the black eschar on the tick bite site and then treated with amoxicillin 500 mg three times a day for 7 days (data unpublished). The eschar surrounded by erythema appeared 24 h after tick removal and the improvement of skin condition was observed after 48 h of antibiotic treatment. The tick was identified as *D. reticulatus* females infected with *R. raoultii.* According to the recommendations of The Polish Society of Epidemiology and Infectious Diseases, the laboratory criteria for rickettsiosis confirmation include the detection of a fourfold increase in antibody titre in sera during the acute and recovery phase of infection or detection of *Rickettsia* DNA in blood/ eschar^61^. In our study, serological and/or molecular tests were not ordered by GP, therefore confirmation of rickettsiosis according to recommendations was not possible.

## Conclusion

This study describes the ticks infestation pattern, *Rickettsia* prevalence and possible medical consequences of *D. reticulatus* feeding on human skin. Information about tick infestation patterns provided by this study should be valuable for predicting the biting location of ticks dependent on species and potentially reducing the transmission of tick-borne pathogens by quick tick removal. The results of this study indicate that even though *D. reticulatus* bite human sporadically, pathogenic rickettsial species have a remarkably high prevalence in this tick species. We have also confirmed the first case of *R. aeschlimannii* infection in *D. reticulatus* female. Noteworthy is that all participants reporting non-specific, flu-like symptoms, including lymphadenopathy, after tick removal were bitten by *D. reticulatus* infected by *R. raoultii,* and in one case rickettsiosis was confirmed by GP based on clinical manifestation (eschar). We can expect that the incidence of tick-borne lymphadenopathy (DEBONEL/TIBOLA) might increase with the reported expansion of the *D. reticulatus* vector into new areas and its growing abundance in Central Europe.

## Materials and methods

### Ethics approval and consent to participate

The Internal Review Board of the Warsaw Medical University was informed about the study protocol (no. AKBE/73/2021). The study protocol followed ethical guidelines of the 2013 Declaration of Helsinki. Informed consent was obtained from all individual participants included in the study.

### Study design

The research reported here was conducted over a 2-year period in 2021 and 2022. The information about our study was disseminated on the University of Warsaw website and on the websites dedicated to medicine, diagnostics, and health care, in social networks, as well as among the university society through email (researchers, students, and administration workers). The study subjects were asked to bring or send their tick(s) to the Department of Parasitology, University of Warsaw in a tightly-sealed, ethanol-filled container within 5 days after removal of the tick(s) from the skin by a physician or the patients themselves. Each patient was included in the study after an informed consent and received information on the aims and the protocol of the study. In case of patients under the legal age of consent (< 18 years old), one of the parents signed the agreement.

### Questionnaires

The study participants were asked to fill out an online questionnaire at enrolment which included questions on the number of tick bite(s), where the tick(s) was/were encountered (urban/rural), the use of chemoprophylaxis and/or protective suits. All the patients were also asked for follow-up after 8 weeks by mail or telephone. The second questionnaire consisted the questions about new tick bites and the person’s general health condition during the past two months, reddening at the bite site, symptoms possibly associated with tick-borne diseases, including *erythema migrans*, medical records from participants who attended general practitioner/infectious diseases specialist appointments for symptoms possibly associated with tick-borne diseases, questions about the results of serological test for Lyme borreliosis (if such were performed) as well as antibiotic treatment within the last 8 weeks. The individuals who reported a second bite within the 8 weeks from first notification and the patients with immunosuppression were excluded from further study.

### Tick collection and identification

The ticks were collected throughout Poland from March to November of each year (2021, 2022). Ticks were morphologically identified in terms of species and developmental stage using a standard taxonomic key^62^. Specimens that could not be identified due to being extensively damaged when being removed from the skin were not included in the study. To confirm morphological identification of *D. reticulatus* nymphs, the fragment of the cytochrome c oxidase subunit I (*cox*1) was amplified and sequenced^63^. The new nucleotide sequence of the *cox1* gene fragment of *D. reticulatus* ticks has been deposited in the GenBank database under accession number OQ947121.

### DNA extraction and PCR analysis

Individual adult ticks were washed in 70% sterile ethanol and then in sterile water to avoid DNA contamination and then homogenised using sterile, stainless steel beads and automatic TissueLyser II (Qiagen, Germany). Genomic DNA from ticks was isolated using the DNeasy Blood & Tissue Kit (Qiagen, Germany) according to the manufacturer’s protocol. Genomic DNA was also used for molecular screening for: (i) *Anaplasma phagocytophilum* through amplification of the fragment of the 16S rDNA^64^; (ii) *Babesia* spp. through amplification of the fragment of the 18S rDNA^65^; (iii) *Borrelia* spirochetes through amplification of the flagellin gene (*flaB*)^66^; (iv) *Rickettsia* spp. through amplification of the fragment of the *gltA* gene^67^ and of the *ompB* gene^68^; and (v) *Neoechrlihia mikurensis* through amplification of the fragment of the *groEL* gene^69^. Negative controls were performed in the absence of template DNA. *Babesia microti* King’s College strain DNA isolated from infected BALB/c mice blood and sequenced *Borrelia afzelii, A. pahocytophilum, R. helvetica* and *N. mikurensis* DNA obtained from infected ticks and humans^3,70,71^ were used as positive controls. PCR products were visualized in 1.5% agarose gels stained with Midori Green Stain (Nippon Genetics Europe, Germany). *Rickettsia*-positive samples from ticks were sequenced by a private company (Genomed S.A., Warszawa, Poland) in both directions. Obtained nucleotide sequences were analysed using BLAST NCBI and MEGA v. 11.0 software^72^ for sequence alignment and species typing using sequences deposited in GenBank NCBI. The new, representative nucleotide sequences of *Rickettsia* species have been deposited in the GenBank database under accession numbers OQ689706-OQ689708 (*gltA*) and OR000446-OR000448 (*ompB*).

### Statistical analysis

Statistical analysis was performed using IBM SPSS Statistics v. 27.0 software (IBM Corp., Armonk, NY, USA). The prevalence of *Rickettsia* infection (percentage of ticks infected) was analysed using maximum likelihood techniques based on log-linear analysis of contingency tables (HILOGLINEAR). For analysis of the prevalence of *Rickettsia* in ticks, we fitted the prevalence of pathogens as a binary factor (infected = 1, uninfected = 0) and then by year (2 levels: 2021–2022), tick stadium (female and male) and type of area (urban and rural). For analysis of the associations between *Rickettsia* DNA detected in ticks or tick species and the patient-reported data at enrolment and after two months, we used the same statistical approach. We fitted the *Rickettsia* prevalence as a binary factor (infected = 1, uninfected = 0) and then by among others self-reported flu-like symptoms (fatigue, headache, loss of appetite, loss of weight, nausea, fever, neck pain, loss of appetite, vertigo, cognitive difficulties, radiating pain, mylagia/ arthralgia, numbness), cutaneous manifestations (local reaction to tick bite) and *erythema migrans* or eschar (‘tache noir’) diagnosed by a general practitioner (GP) or infectious diseases specialist (present and not present). We also fitted the tick species as a binary factor (*I. ricinus* = 1, *D. reticulatus* = 2) and then by sex, immunosuppression, site of tick attachment, number of ticks detached, where the tick was encountered, estimated time of tick attachment, previous tick bites, and use of chemoprophylaxis/ protective suits. P values < 0.05 were considered to be statistically significant.

### Ethics declarations

The Internal Review Board of the Warsaw Medical University was informed about the study protocol (no. AKBE/73/2021). The study protocol followed ethical guidelines of the 2013 Declaration of Helsinki. Informed consent was obtained from all individual participants included in the study.

## Supplementary Information

Supplementary Figure 1.

Supplementary Information 2.

## Data Availability

The datasets used and analysed during this study are available from the corresponding author (RWF) on reasonable request. The new nucleotide sequences of *Rickettsia* species have been deposited in the GenBank database under accession numbers OQ689706-OQ689708 and OR000446-OR000448.
