# Supplementary figures and images for: Rickettsia species in Dermacentor reticulatus ticks feeding on human skin and clinical manifestations of tick-borne infections after tick bite
Source: Sci Rep. 2023 Jun 19;13:9930. doi: 10.1038/s41598-023-37059-3 (PMC10279655; doi:10.1038/s41598-023-37059-3)

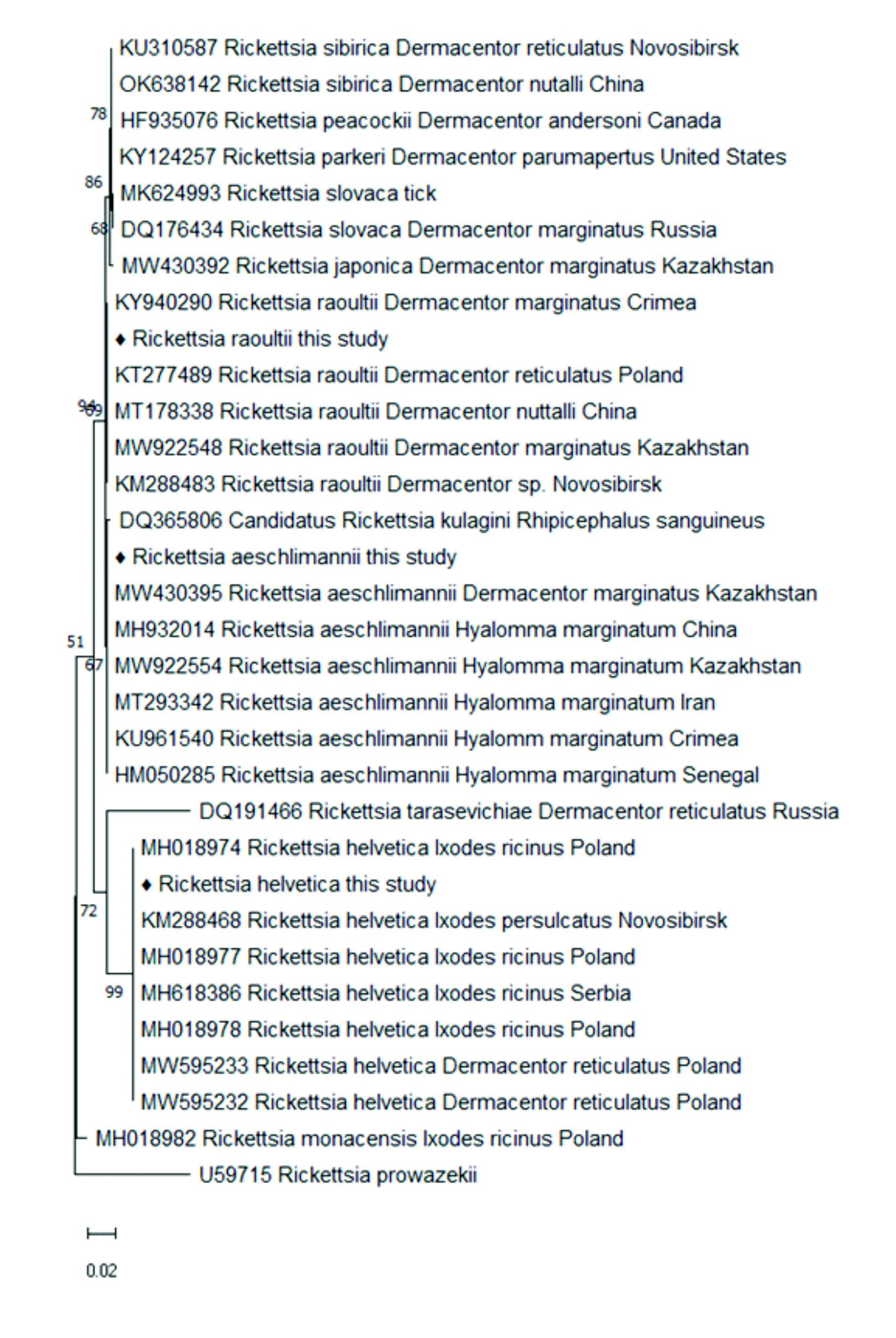

Supplement: Supplementary file 1 — Supplementary Figure 1. [file 41598_2023_37059_MOESM1_ESM.jpg]
