# Supplementary material for: Rickettsia species in Dermacentor reticulatus ticks feeding on human skin and clinical manifestations of tick-borne infections after tick bite
Source: Sci Rep. 2023 Jun 19;13:9930. doi: 10.1038/s41598-023-37059-3 (PMC10279655; doi:10.1038/s41598-023-37059-3)
Supplement: Supplementary file 2 — Supplementary Information 2. [file 41598_2023_37059_MOESM2_ESM.docx]

Supplementary File 1. The phylogenetic tree of *Rickettsia* isolated from different ticks species based on a fragment of the *gltA* gene, was inferred using the Maximum Likelihood method and a Tamura 3-parameter model. The percentage of replicate trees in which the associated taxa clustered together in the bootstrap test (1000 replicates) are shown next to the branches. The analysis involved 32 nucleotide sequences. All positions containing gaps and missing data were eliminated. The nucleotide sequence of *Rickettsia prowazekii* was used as an outgroup. The Rickettsia sequences obtained in this study were marked (♦). Evolutionary analyses were conducted in MEGA 11.0.
